# Problematic Social Situations for Peer-Rejected Students in the First Year of Elementary School

**DOI:** 10.3389/fpsyg.2016.01925

**Published:** 2016-12-15

**Authors:** Luis J. Martín-Antón, María Inés Monjas, Francisco J. García Bacete, Irene Jiménez-Lagares

**Affiliations:** ^1^Department of Psychology, Excellence Research Group GR179 Educational Psychology, University of ValladolidValladolid, Spain; ^2^Department of Developmental, Educational and Social Psychology and Methodology, Jaume I UniversityCastelló de la Plana, Spain; ^3^Department of Developmental and Educational Psychology, University of SevilleSeville, Spain

**Keywords:** peer rejection, peer relations, social status, gender, elementary school

## Abstract

This study examined the social situations that are problematic for peer-rejected students in the first year of elementary school. For this purpose, exploratory and confirmatory factor analyses were conducted on the Taxonomy of Problematic Social Situations for Children (TOPS, Dodge et al., 1985) in 169 rejected pupils, identified from a sample of 1457 first-grade students (ages 5–7) enrolled in 62 classrooms of elementary school. For each rejected student, another student of average sociometric status of the same gender was selected at random from the same classroom (*n*_average_ = 169). The model for the rejected students showed a good fit, and was also invariant in the group of average students. Four types of situations were identified in which rejected students have significantly more difficulties than average students. They are, in descending order: (a) respect for authority and rules, (b) being disadvantaged, (c) prosocial and empathic behavior, and (d) response to own success. Rejected boys have more problems in situations of prosociability and empathy than girls. The implications concerning the design of specific programs to prevent and reduce early childhood rejection in the classroom are discussed.

## Introduction

Peer interactions in childhood are one of the pillars of child development, as they are the basis for building future relationships (Gifford-Smith and Brownell, 2003; Green et al., 2008). Among them, the relationship with classmates is of particular interest because children maintain constant contact at school and in extracurricular situations, and currently, also in virtual environments (Gallagher, 2005). Thus, the classroom becomes the context for academic learning, but also the basic framework of coexistence and relationship among students, enabling the implementation of important emotional and social skills (Mikame et al., 2010; Comellas, 2013). Peer exchanges contribute to the development of significant cognitive and socio-emotional achievements (Ladd, 2005; Rose-Krasnor and Denham, 2009) and hence, to school adaptation (Gifford-Smith and Brownell, 2003). Inadequate or deficient relationships during childhood can lead to diverse problems later on (Hartup, 1989; van Ijzendoorn, 2005; Hay et al., 2009; Pérez-Fuentes et al., 2016).

These issues are especially important for children between 5 and 6 years who begin compulsory schooling. The first year of elementary school is a stressful situation because students face the new academic and coping challenges of greater teacher and school demands (Settanni et al., 2015). At the same time, it poses difficult interpersonal challenges arising from peer group entry, which will involve the implementation of new and more complex emotional skills (Ladd, 2005; Durlak et al., 2011). Being accepted and loved by their classmates, having friendly and satisfactory relationships with others, being integrated and participating actively in the group, and building dyadic relationships and friendships with peers are some of the aspects that children must achieve to attain optimal emotional, cognitive, and social development (Merrell and Gueldner, 2010).

It is also relevant for children to maintain warm relationship with the authority figure represented by the teaching staff (Birch and Ladd, 1997; Baker, 2006; Cadima et al., 2010; Koomen et al., 2012; Fraire et al., 2013; García Bacete et al., 2014; Lee and Bierman, 2016). Teachers are highly involved in the social dynamics of the classroom and in the specific aspects of vulnerable students' relationships (Kiuru et al., 2012). Rudasill and Rimm-Kaufman (2009) point out the importance of the frequency of teacher-student interactions. The quality of this interaction plays an important role in children's personal, social, and academic success, especially in children who are at risk of failure (Hamre and Pianta, 2005; García Bacete et al., 2014; Bush et al., 2015). The quality of these relationships is usually stable (Pianta and Stuhlman, 2004) and could depend on the teachers' gender and that of their students (Quaglia et al., 2013).

In fact, most children achieve positive relationships with their peers. Some children have a privileged social position: they are the preferred students, highly valued by their peers. Others simply get along well with others and have a few friends. However, there are some children who, for various reasons, do not fit in the group and are passively or actively rejected and excluded by their peers. These are the children with a rejected sociometric status. The identification of the sociometric type is usually done through sociometric strategies, based on collecting the relationship preferences of the classmates of each student (Cillessen, 2009; García Bacete and González Álvarez, 2010). According to the works of Coie et al. (1982), depending on the number of positive and negative nominations received by each student of the group, five sociometric types have been established: in addition to the popular and rejected status, there are the average, neglected, and controversial status.

In recent decades, developmental research has devoted considerable attention to the phenomenon of peer rejection, noting the harmful consequences for the socio-emotional, cognitive, and academic development of the rejected students (Gifford-Smith and Brownell, 2003; Bierman, 2004; Sandstrom and Zakriski, 2004; Asher and McDonald, 2009; Wentzel, 2009). Interest in rejection derives from its high incidence—between 10 and 15% of the students are rejected by their peers (García Bacete et al., 2008; McKown et al., 2011)—from its negative consequences—as well as involving important suffering by the rejected child, it predicts various psychological problems, academic failure, and dropout (Mayeux et al., 2007)—and from the stability and persistence of its effects (Coie and Dodge, 1983; Coie, 1990; Cillessen et al., 2000; Jiang and Cillessen, 2005).

Although the population of rejected students is very heterogeneous, numerous studies have tried to establish a profile associated with various behavioral, cognitive, and emotional correlates. Thus, Bierman (2004) points out that rejected students share some of the four following behavioral patterns; (a) low rates of sociability, orientation toward others, and prosocial behavior (low empathy, poor cooperative behaviors); (b) high aggression and disruptive behavior, which tends to predict situations of rejection in subsequent courses (Bierman et al., 2014); (c) high levels of immature behavior and lack of attention; and (d) social anxiety and avoidance behaviors. Along with these features, other characteristics emerge, such as low emotional self-regulation and difficulties to perceive, understand, and regulate emotions (Southam-Gerow and Kendall, 2002), difficulties to understand situational demands and interpret social signals, and in perspective-taking (White and Kistner, 2011). They also have social information processing biases, for example, when interpreting the reasons for others' behavior, rejected children frequently make hostile attributions to their classmates' behavior, especially in ambiguous situations that they interpret erroneously (Dodge et al., 2003; Dirks et al., 2007; Lansford et al., 2010). All this leads them to respond to the situation maladaptively.

Therefore, it could be argued that rejected children are socially less competent than their more valued peers. We should therefore clarify what is meant by being less competent. The approach of Asher and McDonald (2009) focuses on the behavior emitted by the child in response to specific social situations. They point out that problems in relationships do not necessarily appear in all social situations, but rather in some very specific situations that pose a problem for the child. Social competence is thus not considered so much as a general trait, but as the ability to respond adequately to different circumstances (Asher et al., 2012).

Asher and McDonald (2009) presented a list of 40 social situations, among which are peer group entry, ambiguous provocation, seeking, or offering help, or conflict management. Dodge et al. (1985) found that the greatest differences between rejected and unrejected children occurred in the Response to Provocation and in Teacher Expectations. Parker and Asher (1987) demonstrated that, for socially incompetent and aggressive children, peer group entry and knowing how to react to provocations were the most difficult social situations for them within the context of peer relationships. A similar response pattern is observed in all of these situations: compared to accepted children, children with low acceptance tend to emit more aggressive responses and fewer socially sophisticated responses (Asher et al., 2012).

Observational laboratory studies and vignettes have frequently been used to appraise social situations. The use of peers or teachers as informants is much less common (Asher and McDonald, 2009). However, teachers are quite familiar with their students' behavior and difficulties, and therefore, they have frequently been used to appraise various aspects of social competence and, in particular, rejected students' difficulties. It has been found that teachers are good informants (Pouwels et al., 2016), providing information that correlates highly with that provided by peers (McKown et al., 2011). These correlations are higher in studies of both sexes, and in studies carried out at school instead of in the laboratory (Renk and Phares, 2004).

## Taxonomy of problematic social situations for children

Dodge et al. (1985) proposed the Taxonomy of Problematic Social Situations for Children (TOPS) based on the identification of 64 situations by 50 teachers from first to fifth grade, grouped into eight categories. After a subsequent refinement, the list was reduced to 44 situations. To assess its psychometric properties, it was applied to a group of 45 rejected students and 39 average children from a general sample of 620 students of 23 classrooms from second to fourth grade (7–10 years old). The categories were thus reduced to six: Peer Group Entry, Response to Provocation, Response to Failure, Response to Success, Social Expectations, and Teacher Expectations.

The TOPS is a versatile instrument. Several studies have shown its effectiveness in different areas, for example, the identification of rejection and related situations at school (Nangle et al., 1994). Walker et al. (2002) found that three of the dimensions especially discriminate differences in social competence: Peer Group Entry, Peer Social Expectations, and Response to Provocation, and the last one differentiates intentional and ambiguous situations. The authors also found that girls are more competent in prosociality whereas boys have higher rates of aggressive responses. van Manen (2006) used the TOPS to assess the effectiveness of an intervention to reduce children's aggressive behavior. It has also been used in clinical settings, in studies of children with high levels of prenatal alcohol exposure (Timler, 2000). This author also compared the dimensions of the TOPS in children with and without language disorders, finding in the latter greater difficulties responding to provocation (Timler, 2008). Shah and Morgan (1996) related the TOPS to depressive symptoms in adolescents, reporting high discriminant power. Green et al. (2008) used this taxonomy, among others, to categorize the response to stories concerning the use of prosocial-assertive, passive, and coercive strategies in 6-year-old students.

Other studies have determined the structure factor of the TOPS, proposing short versions. Matthys et al. (2001) obtained a short version of the TOPS from exploratory factor analysis (EFA) and confirmatory factor analysis (CFA) of the original scale in a sample of 652 students from first to sixth grade of elementary school, with appropriate psychometric properties. It consists of 18 items grouped into four oblique factors that explain 71.6% of the variance: Being Disadvantaged (consisting of 6 items from the factors Peer Group Entry and Response to Provocation from the original taxonomy), Coping with Competition (with 4 items from Response to Failure and Response to Success from the original taxonomy), Social Expectations of Peers (4 items), and Teacher Expectations (4 items), which correspond, respectively, to the fifth and sixth factor of the original taxonomy. Boys have more difficulties than girls in all the factors. With regard to the grade, only differences in Being Disadvantaged were found, with a decrease in difficulties as the grade advanced. However, they found no interaction between gender and grade. It must be borne in mind that out of the entire sample, 119 were first graders of elementary school and from all the sociometric status. The Taxonomy of Problematic Social Situations-Adolescent Self-report Version (TOPS-A; van der Helm et al., 2013) was developed from the data of a sample of 128 young people in secure institutional and correctional youth care. It is made up of 22 items grouped into four factors: Disadvantage (8 items), Competition (5 items), Accepting/giving help (3 items), and Accepting Authority (6 items).

Other authors have developed versions for other age groups. For example, the Preschool Taxonomy of Problem Situations (PTOPS; Blankemeyer et al., 2002) was applied it to a sample of 42 abused preschoolers aged 3–5. It comprises 60 items grouped into eight factors: Peer Group Entry, Response to Provocation, Response to Failure, Response to Success, Social Expectations, Teacher Expectations, Reactive Aggression, and Proactive Aggression.

Summing up, a large part of the studies are promising, although they were conducted with small samples (Nangle et al., 1994; Barn, 2014), with clinical characteristics such as childhood abuse, juvenile delinquency, personality problems, etc. (Blankemeyer et al., 2002; van der Helm et al., 2013), considering the entire stage of elementary school and all the sociometric status (Matthys et al., 2001), or with adolescents suffering chronic rejection, the consequences of which have marked their socio-emotional development.

## Aims of the present study

Considering the above-mentioned issues, the present study aims to identify, in a large sample, social situations that are specifically problematic for peer-rejected students at a crucial moment of their development such as the beginning of their compulsory schooling, as it will not consider situations that may not be very relevant at this age or for other sociometric status. In addition, it is expected that the degree to which these social situations are problematic will be related to inappropriate social behavior, and even antisocial behavior, which would provide an adequate indicator of convergent validity.

Finally, we intend to verify whether the identified model is also applicable to students with average sociometric status, comparing their results with those of the rejected students. Knowing the degree to which rejected students have more problems in social situations than average students, while taking gender into account, can provide valuable information to implement specific actions designed to prevent and reduce peer rejection at early ages.

## Methods

### Participants

We started with an initial sample *N* = 1457 students (730 female) and their 62 teachers from 62 first-grade classrooms of urban public schools in four cities of Spain (37% from Castellón, 20% from Palma de Mallorca, 22% from Seville, and 21% from Valladolid). The number of students per classroom ranged between 18 and 27 (*M* = 23.5, *SD* = 2.15). They were between 5 and 7 years of age (*M* = 6.41, *SD* = 0.37), although 98% (*n* = 1430) were of the normative age corresponding to first grade. Of the rest, *n* = 26 children were 1 year older (because they were repeaters) and one child was 1 year younger (due to academic acceleration). The cultural and demographic characteristics of the schools are equivalent, with a similar number of students as in other countries and subcultures. The students from other countries represent 12.3% and mainly come from South America and Eastern Europe.

A sociometric procedure identified peer-rejected students, who represent 12.4% (*n* = 181), ranging from one to five rejected students per classroom (*M* = 2.91, *SD* = 0.99). We eliminated 12 subjects because they presented more than 50% school absenteeism, so their teachers did not have enough data to make an accurate assessment. As a result, the final sample of rejected students was *n*_peerrejected_ = 169 (109 males). As comparison sample, for each rejected student, we randomly selected a student with average sociometric status, from their same classroom and gender (*n*_average_ = 169, 109 males).

The present study was conducted in accordance with the 1964 Helsinki declaration and its later amendments or comparable ethical standards, with the approval of the management board of schools, the educational inspection services, the Department of Education of the Regional Government of Valencia (Spain), the Childhood Observatory of the Regional Government of Andalusia (Spain), the Socio-Educational Institute Foundation s'Estel of the Government of the Balearic Islands (Spain); and the Observatory School Coexistence of the Autonomous Government of Castilla y León (Spain). Participation in the study was voluntary. All subjects gave written informed consent in accordance with the Declaration of Helsinki.

### Measures

#### Taxonomy of problematic social situations for children (TOPS; Dodge et al., 1985)

This is a 44-item Likert scale on which the teachers rate the response that each student displays in different social situations, ranging from 1 (*never poses a problem*) to 5 (*almost always represents a problem*). Items are grouped into six factors with high internal consistency (total α = 0. 98): (a) Peer Group Entry (5 items, α = 0.95), (b) Response to Provocation (10 items, α = 0.97), (c) Response to Failure (9 items, α = 0.95), (d) Response to Success (3 items, α = 0.89), (e) Social Expectations (11 items, α = 0.94), and (f) Teacher Expectations (6 items, α = 0.95). Like Matthys et al. (2001), we eliminated Item 31 (“when this child is seated at lunch with a group of peers and a teacher is not nearby”) because only 25% of the students habitually had lunch at school, and also the teachers were not usually present, so they did not have enough information to assess this item.

#### Peer nominations sociometric questionnaire

This questionnaire can be applied individually or collectively, and each student chooses classmates, in a prioritized and reasoned fashion, based on two blocks of questions that can be applied together or separately. The first block contains two questions relating to the acceptance and rejection of his or her classmates (“who do you like to be with the most?” and “who do you like to be with the least?”). The second block also contains two questions about their perception of their acceptance and rejection by others (“The classmate/classmates that he/she believes like to be with him/her” and “the classmate/classmates that he/she believes do not like to be with him/her”). Normally, one, three, or unlimited nominations are allowed. The data were analyzed with the Sociomet software (González and García Bacete, 2010), according to an adaptation of the probabilistic procedure by Newcomb and Bukowski (1983). It classifies students into the different groups proposed by Coie et al. (1982): rejected, popular, controversial, neglected, and average.

#### School social behavior scales (SSBS-2; Merrell, 2002, translated into spanish by Salazar and Caballo, 2006)

This scale measures the teaching staff's perception of the students' social behavior in the school environment. It has 64 items rated on a 5-point Likert scale ranging from 1 (*never*) to 5 (*often*), distributed in two scales of 32 items each, and six subscales, with good test-retest reliability, inter-evaluator agreement, high internal consistency, and fit indices in the confirmatory factor analysis (Crowley and Merrell, 2003). The A Scale, Social Competence assesses Peer relations (14 items, α = 0.97), Self-management/compliance (10 items, α = 0.95), and Academic behavior (8 items, α = 0.95). The B Scale, Antisocial Behavior, measures Hostile/irritable behavior (14 items, α = 0.96), Antisocial/aggressive (10 items, α = 0.93), and Defiant/disruptive (8 items, α = 0.91).

### Procedure

After reaching agreements with the schools and obtaining the families' authorizations, the sociometric questionnaire was administered to the 1457 students, in the form of individual interviews, carried out by several trained assessors (hired or postgraduate psychology or educational psychology research collaborators). An unlimited number of nominations was allowed, by presenting the classmate's photo, where, in addition to the photo, his/her name or list number appeared. The administration was performed 2 months after start of the school year and it lasted 3 weeks, allowing all the students who regularly attended school to participate. From the acceptance and rejection nominations, analyzed with the Sociomet software, the following sociometric distribution was established: (a) 68.5% (*n* = 997) average students, (b) 13.2% (*n* = 192) popular students, (c) 12.4% (*n* = 181) rejected students, (d) 4.5% (*n* = 66) neglected students, and (e) 1.4% (*n* = 21) controversial students. As commented above, we eliminated 12 cases due to high absenteeism, leaving a total of 169 rejected students (109 males). This distribution is consistent with previous studies that estimate between 10 and 15% of rejected students, and of these, between 65 and 75% are males. Subsequently, an average-status, same-gender student was randomly assigned for each rejected student, with the condition that he/she must be from the same classroom, thereby forming, together with the rejected students, a total sample of 338 students. The teachers were asked to fill in the TOPS and the SSBS-2 with reference to these students, without knowing who were rejected or average students.

### Data analysis

Firstly, we performed EFA to detect the specific internal structure of the profile of rejected students at this age. Previously, we tested the assumption of multivariate normality through Mardia's coefficient, which should not exceed the value of 3 to assume multivariate normality (Mardia, 1970). We applied principal component analysis (unbiased for not fulfilling normality), selecting components with eigenvalues greater than 1, with two rotation methods to ensure a better fit: one based on an orthogonal Varimax model, and another oblique Promin model (Lorenzo-Seva, 1999), based on polychoric correlations, considering the items as ordinal. Calculations were done with the statistical software FACTOR (Lorenzo-Seva and Ferrando, 2013) version 10.1. In addition to calculating the solution from the polychoric correlation matrix, this program also provides data for multivariate goodness of fit.

We eliminated all items with factor loadings under 0.40, items that loaded significantly on more than one factor, and factors that did not have at least three indicators for each latent variable, because fewer indicators lead to identification and convergence difficulties (Lomax, 1982; Bentler and Chou, 1987; Anderson and Gerbing, 1988). We also calculated the internal consistency alpha coefficient, the factor simplicity indexes through Bentler's simplicity index (S, Bentler, 1977) and the loading simplicity index (LS; Lorenzo-Seva, 2003), as well as Cronbach's standardized alpha and the root mean square of residuals (RMSR), using the criterion proposed by Kelley (Kelley, 1935; Harman, 1962).

Next, we tested the structure factor found with CFA, comparing it to three alternative models. As the variables were ordinal and the multivariate normality assumption was not met, we used robust maximum-likelihood estimation (Satorra-Bentler scaled statistics or S-B χ^2^, *p* > 0.05), although it is highly conditioned by sample size. Therefore, we complemented it with other indices that assess model fit (Bollen and Long, 1993). Among them, we used the relative chi square index (χ^2^/*df*), whose values should be lower than 2 or 3 (Ullman, 2007; Kline, 2010), although a value lower than 5 can also be considered acceptable (Schumacker and Lomax, 2004); the comparative fit index (CFI > 0.95; Bentler, 1990); the normed fit index (NFI > 0.95; Bentler and Bonett, 1980; Hu and Bentler, 1999); the non-normed fit index (NNFI > 0.90; Hu and Bentler, 1999); the robust root mean-square error of approximation (RMSEA < 0.08; Hu and Bentler, 1999), and the Akaike information criterion (AIC; Akaike, 1974), which is useful to compare models, considering the criterion with the lowest value as the most adequate. We also checked the adequacy of the composite reliability (CR > 0.70), the convergent (average variance extracted, AVE > 0.50) and discriminant validity, using the variance extracted test, which postulates that the AVE of the related factors must be higher than its squared correlation (Fornell and Larcker, 1981; Netemeyer et al., 1990). To estimate convergent validity, we calculated Pearson correlations between the TOPS factors and the six SSBS-2 subscales.

To determine whether the model is also valid for a sample of different psychometric characteristics, we studied the configural, metric, scalar, and factor mean invariance through multigroup analysis (group of rejected and group of average students), with the Satorra-Bentler scaled chi square difference test (Satorra and Bentler, 2001), using the program developed by Crawford and Henry (2003). We adopted the criterion of Cheung and Rensvold (2002), calculated as the difference between the CFI values, and considering that invariance can be accepted if this difference is less than or equal to 0.01 in favor of less restrictive model (ΔCFI_unconstrained_ − ΔCFI_constrained_ ≤ 0.01). These analyses were conducted with the computer program EQS 6.2 (Build 107).

Finally, two-way multivariate analysis of variance (MANOVA) was applied to determine possible differences in sociometric status and gender. Partial eta-squared effect sizes are presented: 0.01 < $\eta_{p}^{2}$ < 0.05 is considered a small effect, 0.06 < $\eta_{p}^{2}$ < 0.13 is considered a medium effect, and $\eta_{p}^{2}$ > 0.14 is considered a large effect (Cohen, 1988). Inter-subject effects were analyzed in order to determine which variables were significantly different. For paired comparisons, the *t*-test for two independent groups was used, including Cohen's *d* effect size (Cohen, 1988), considering: *d* = 0.20 small, *d* = 0.50 medium, and *d* = 0.80 large effect size. For this purpose, we used the statistical package IBM SPSS Statistics, version 22 (2013). All statistical analyses used a 95% confidence level.

## Results

### Exploratory factor analysis (EFA)

Mardia's coefficient was 18.81, so the assumption of normal multivariate is violated, which is to be expected when working with categorical variables even if they are considered ordinal. However, the skewness or kurtosis values were within normal parameters, as none of the items presented values higher than 2 or 7, respectively (West et al., 1995), as shown in Table 1. The item to item correlations were low to high, ranging from 0.14 to 0.86. Were found only four correlations above a 0.70 level.

**Table 1:** **Polychoric Correlation Matrix and Descriptive Statistics of the Social Situations (*n* = 169)**.

**Social Situations**	**3**	**6**	**12**	**14**	**16**	**17**	**19**	**24**	**27**	**28**	**30**	**33**	**36**	**37**	**38**	**39**	**43**
**CORRELATIONS**																	
3. The student has won a game	—																
6. They insult the student	0.21	—															
12. The student plays a game better	0.68	0.17	—														
14. The student does a better task	0.68	0.17	0.82	—													
16. They do not return the student's belongings	0.20	0.61	0.19	0.16	—												
17. They exclude the student from a game	0.40	0.52	0.34	0.33	0.52	—											
19. They accidentally break the student's toy	0.28	0.67	0.21	0.28	0.69	0.59	—										
24. They provoke the student accidentally	0.29	0.72	0.22	0.22	0.64	0.60	0.67	—									
27. The teacher speaks to the whole class	0.40	0.46	0.34	0.32	0.47	0.34	0.45	0.49	—								
28. The student is in the row	0.34	0.54	0.33	0.32	0.50	0.44	0.49	0.61	0.67	—							
30. In class without the teacher	0.33	0.31	0.39	0.32	0.28	0.23	0.21	0.35	0.57	0.60	—						
33. Others are interested in the student	0.29	0.27	0.27	0.31	0.30	0.14	0.32	0.33	0.33	0.32	0.26	—					
36. They show their anger at the student	0.39	0.54	0.29	0.29	0.59	0.61	0.29	0.63	0.43	0.50	0.21	0.31	—				
37. They expect the student's praise	0.44	0.30	0.50	0.51	0.31	0.32	0.39	0.39	0.38	0.36	0.21	0.46	0.46	—			
38. They expect the student's comfort	0.38	0.23	0.48	0.42	0.32	0.24	0.37	0.32	0.36	0.31	0.18	0.46	0.44	0.86	—		
39. They expect the student's thanks	0.37	0.17	0.43	0.40	0.25	0.19	0.30	0.25	0.29	0.28	0.22	0.47	0.34	0.70	0.79	—	
43. The student must ask for help	0.30	0.26	0.40	0.34	0.31	0.20	0.25	0.26	0.41	0.31	0.25	0.56	0.32	0.58	0.57	0.57	—
**DESCRIPTIVE STATISTICS**																	
*M*	1.97	3.49	1.82	1.70	3.30	3.08	3.12	3.09	2.73	2.96	2.46	2.05	2.93	2.14	2.13	2.14	2.33
*SD*	0.94	1.13	0.91	0.86	1.13	1.05	1.17	1.16	1.28	1.23	1.29	0.91	1.13	0.99	1.00	1.05	1.02
Skewness	0.88	−0.24	1.13	1.31	−0.04	0.02	0.01	−0.06	0.30	0.13	0.60	0.86	0.13	0.60	0.88	0.87	0.55
Kurtosis	0.39	−0.77	1.09	1.72	−0.83	−0.72	−0.81	−0.78	−0.85	−0.90	−0.63	0.59	−0.77	−0.30	0.49	0.43	−0.30

The data were suitable for using EFA, as indicated by the of Kaiser-Meyer-Olkin index (KMO = 0.89) and Bartlett's sphericity test, $\chi_{\left(136\right)}^{2}$ = 1880.80, *p* = < 0.001. The best rotated solution was produced with the Promin method, with four related factors, explaining 73.71% of the variance, with high values in the item factor loadings (see Table 2). Specifically, we obtained (see Spanish items in Appendix): (a) Being Disadvantaged, consisting of 6 items that explain 43.18% of the variance, with internal consistency of α = 0.91. This factor refers to situations in which the child gets damage from their peers (e.g., “when peers call this child a bad name”); (b) Respect for Authority and Rules, with 3 items, explaining 14.51% of the variance, and α = 0.87 (e.g., “when this child is standing in line with peers and must wait a long time”); (c) Response to Own Success, also with 3 items, explaining 8.95%, and α = 0.88 (e.g., “when this child has won a game against a peer”); and (d) Prosocial and Empathic Behavior with 5 items, explaining 7.07% of the variance, and α = 0.83 (e.g., “when a peer is troubled, worried or upset and needs comfort from this child”).

**Table 2:** **Summary of Exploratory and Confirmatory Factor Analysis Results for Social Situations (*n* = 169)**.

**Social Situations**	**Factor loadings for EFA**					**Factor loadings for CFA**			
	**F_1_**	**F_2_**	**F_3_**	**F_4_**	***h*^2^**	**F_1_ St. Est. (*R*^2^)**	**F_2_ St. Est. (*R*^2^)**	**F_3_ St. Est. (*R*^2^)**	**F_4_ St. Est. (*R*^2^)**
**F_1_: BEING DISADVANTAGED**									
6. They insult the student	**0.78**	0.18	−0.18	−0.03	0.70	0.80 (0.65)	–	–	–
16. They do not return the student's belongings	**0.77**	0.09	−0.22	0.11	0.69	0.82 (0.68)	–	–	–
17. They exclude the student from a game	**0.88**	−0.13	0.31	−0.27	0.71	0.71 (0.51)	–	–	–
19. They accidentally break the student's toy	**0.88**	0.07	−0.08	0.08	0.74	0.84 (0.71)	–	–	–
24. They provoke the student accidentally	**0.80**	0.16	−0.11	0.01	0.76	0.86 (0.75)	–	–	–
36. They show their anger at the student	**0.79**	−0.12	0.06	0.12	0.69	0.77 (0.60)	–	–	–
**F_2_: RESPECT FOR AUTHORITY AND THE RULES**									
27. The teacher speaks to the whole class	0.11	**0.72**	0.02	0.12	0.71	–	0.80 (0.64)	–	–
28. The student is in the row	0.28	**0.68**	0.01	−0.01	0.75	–	0.90 (0.81)	–	–
30. In class without the teacher	−0.21	**0.96**	0.16	−0.08	0.79	–	0.69 (0.48)	–	–
**F_3_: RESPONSE TO OWN SUCCESS**									
3. The student has won a game	0.13	0.12	**0.82**	−0.11	0.74	–	–	0.75 (0.56)	–
12. The student plays a game better	−0.04	0.15	**0.87**	0.01	0.84	–	–	0.93 (0.86)	–
14. The student does a better task	0.02	0.09	**0.88**	−0.02	0.82	–	–	0.88 (0.78)	–
**F_4_: PROSOCIAL AND EMPATHIC BEHAVIOR**									
33. Others are interested in the student	−0.08	0.24	−0.16	**0.72**	0.54	–	–	–	0.57 (0.33)
37. They expect the student's praise	0.15	−0.14	0.18	**0.76**	0.80	–	–	–	0.87 (0.75)
38. They expect the student's comfort	0.08	−0.17	0.09	**0.88**	0.83	–	–	–	0.93 (0.86)
39. They expect the student's thanks	−0.06	−0.09	0.07	**0.88**	0.76	–	–	–	0.87 (0.75)
43. The student must ask for help	−0.12	0.17	−0.07	**0.81**	0.65	–	–	–	0.70 (0.49)

*St. Est., Standardized Estimations. EFA factor loadings over 0.40 appear in bold*.

The factor simplicity indices, as well as the overall reliability and fit indices of the model are very high: *S* = 0.9811 (P_100_) and *LS* = 0.4853 (P_100_); standardized Cronbach's α = 0.92; and RMSR = 0.0459 (Kelly's criterion < 0.0772).

### Confirmatory factor analysis (CFA)

The indices showed an excellent fit, S-B $\chi_{\left(113\right)}^{2}$ = 132.41, *p* = 0.101; S-B χ^2^/*df* = 1.17, CFI = 0.99, NFI = 0.97, NNFI = 0.99, RMSEA = 0.032, 90% CI [0.000, 0.052]. The *Lagrange multipliers contrast* and the Wald test did not indicate significant improvements, so it was not necessary to respecify. The composite reliability was high (0.92) and both composite reliability (CR_F1_ = 0.91, CR_F2_ = 0.84, CR_F3_ = 0.89, CR_F4_ = 0.89) and average variance extracted (AVE_F1_ = 0.648, AVE_F2_ = 0.643, AVE_F3_ = 0.730, AVE_F4_ = 0.636) exceeded the criterion values to be considered appropriate. Discriminant validity was good, as in all cases the extracted variance test was exceeded (Table 3).

**Table 3:** **Composite reliability indices, variance extracted indices, and correlations between the factors of the rejected students**.

**Factor**	**CR**	**AVE**	***r* (*r*^2^)**		
			**F_1_**	**F_2_**	**F_3_**
F_1_: Being Disadvantaged	0.91	0.648			
F_2_: Respect for Authority and the Rules	0.84	0.643	0.73 (0.53)		
F_3_: Response to Own Success	0.89	0.730	0.31 (0.01)	0.42 (0.18)	
F_4_: Prosocial and Empathic Behavior	0.89	0.636	0.46 (0.21)	0.43 (0.19)	0.57 (0.33)

*CR, Composite Reliability; AVE, Average Variance Extracted*.

Given that there was a significant reduction of items and sample specificity, we compared the fit of the previous model with other alternatives: (a) four orthogonal (independent) factors; (b) a hierarchical model in which the four factors are explained by a second-order factor; and (c) a univariate model in which all the items explain a single factor. The fit indices of the four models are shown in Table 4. It can be observed that the fit was not satisfactory in any of the three proposed alternative models, which also presented a higher AIC index.

**Table 4:** **Goodness-of-fit indexes of the four possible models**.

**Model**	**S-B χ^2^**	***df***	**S-B χ^2^/*df***	**CFI**	**NFI**	**NNFI**	**RMSEA, 90% [CI]**	**AIC**
Four oblique factors	132.41	133	1.17	0.99	0.97	0.99	0.032, [0.000, 0.052]	−93.54
Four orthogonal factors	276.05^***^	119	2.32	0.96	0.93	0.96	0.089, [0.075, 0.102]	38.05
Hierarchical model	1197.09^***^	115	10.41	0.84	0.82	0.82	0.182, [0.169, 0.193]	967.08
Unifactorial model	1063.28^***^	119	8.93	0.77	0.75	0.74	0.217, [0.205, 0.229]	825.28

*S-B χ^2^, Satorra-Bentler Scaled Statistics; CFI, Comparative Fit Index; NFI, Normed Fit Index; NNFI, Non-Normed Fit Index; RMSEA, Root Mean-Square Error of Approximation; CI, Confidence Interval; AIC, Akaike Information Criterion*.

*** *p < 0.001*.

### Convergent validity

To estimate convergent validity, we correlated the problematic situations factors with the social competence or antisocial behavior displayed in the school setting as measured by the SSBS-2 of Merrell (2002). These data are shown in Table 5. All the correlations were significant, although with a different sign and degree. Initially, the most notable correlations were the high positive correlation between Being Disadvantaged and Antisocial Behavior, and the negative correlation between Being Disadvantaged and Self-management/Compliance. Respect for Authority and Rules had a high positive correlation with Antisocial Behavior and a high negative correlation with Self-management/Compliance. Response to Own Success had the lowest, albeit significant, negative correlation with Social Competence, and a positive correlation with Antisocial Behavior.

**Table 5:** **Correlations between the problematic situations factors and the SSBS-2 (*n* = 169)**.

**Measure**	**PR**	**S-M/C**	**AB**	**H/I**	**A/A**	**D/D**
Being Disadvantaged	−0.33^***^	−0.59^***^	−0.35^***^	0.69^***^	0.66^***^	0.71^***^
Respect for Authority and Rules	−0.40^***^	−0.67^***^	−0.44^***^	0.58^***^	0.61^***^	0.74^***^
Response to Own Success	−0.26^***^	−0.36^***^	−0.23^***^	0.47^***^	0.46^***^	0.43^***^
Prosocial and Empathic Behavior	−0.40^***^	−0.43^***^	−0.34^***^	0.48^***^	0.48^***^	0.51^***^

*PR, Peer Relations; S-M/C, Self-Management/Compliance; AB, Academic Behavior; H/I, Hostile/Irritable; A/A, Antisocial/Aggressive; D/D, Defiant/Disruptive*.

*** *p < 0.001*.

### Invariance analysis between rejected and average students

Firstly, the fit indices of the model applied to the sample of students with average sociometric status were verified. These fit indices were adequate, similar to those obtained with the rejected students, S-B $\chi_{\left(113\right)}^{2}$ = 127.82, *p* = 0.161; S-B χ^2^/*df* = 1.13, CFI = 0.99, NFI = 0.97, NNFI = 0.99, RMSEA = 0.028, 90% CI [0.000, 0.050]. The composite reliability and AVE presented adequate values (see Table 6), as did discriminant validity.

**Table 6:** **Composite reliability indices, extracted variance indices, and correlations between the factors of average students**.

**Factor**	**CR**	**AVE**	***r* (*r*^2^)**		
			**F_1_**	**F_2_**	**F_3_**
F_1_: Being Disadvantaged	0.90	0.608			
F_2_: Respect for Authority and the Rules	0.79	0.560	0.37 (0.13)		
F_3_: Response to Own Success	0.79	0.546	0.21 (0.05)	0.27 (0.07)	
F_4_: Prosocial and Empathic Behavior	0.88	0.594	0.28 (0.08)	0.30 (0.09)	0.30 (0.09)

*CR, Composite Reliability; AVE, Average Variance Extracted*.

We subsequently analyzed the factorial invariance, conducting a multigroup analysis without any restrictions (see Table 7). The configural model will serve as a baseline for the comparison with the nested models on which successive restrictions will be imposed. The fit indices of this model were also acceptable. If we restrict the factor loadings of the items of this model (weak invariance), we obtain acceptable data. The difference between the CFI values of the models was acceptable (ΔCFI = 0.00) and the Satorra-Bentler scaled difference test was nonsignificant, $\chi_{\left(13\right)}^{2}$ = 16.11, *p* = 0.243, showing that metric invariance was fulfilled. The following nested model adds to the former models the restriction of the intercepts, in order to determine possible scalar invariance. The result was not satisfactory. The *Lagrange multipliers contrast* suggested freeing the equality restrictions of the intercepts of Items 3, 14, and 38. In this corrected model, the difference between the models of the CFI value was acceptable (ΔCFI = −0.01) and the Satorra-Bentler scaled difference test was nonsignificant, $\chi_{\left(19\right)}^{2}$ = 26.66, *p* = 0.113), showing that partial strong variance is met. We subsequently determined possible differences in the means of the latent factors. In this case, we expected that the invariance assumption would not be met, given that, theoretically, the two groups should obtain different outcomes, as, in fact, occurred. The fit indices of this model were not satisfactory. The difference in the CFI value far exceeded the criterion value, ΔCFI = −0.25, and the Satorra-Bentler scaled difference test was significant, $\chi_{\left(23\right)}^{2}$ = 85.56, *p* = < 0.001. As a result, equality of means of the latent factors could not be assumed.

**Table 7:** **Model summary for multi-group test of measurement invariance**.

**Model**	**S-B χ^2^**	***df***	**S-B χ^2^/*df***	**CFI**	**NFI**	**NNFI**	**RMSEA, 90% [CI]**
Configural	270.54^*^	226	1.20	0.99	0.97	0.99	0.034, [0.014, 0.049]
Full metric	286.94^*^	239	1.20	0.99	0.97	0.99	0.035, [0.015, 0.048]
Full scalar	386.50^***^	248	1.56	0.80	0.62	0.76	0.059, [0.048, 0.070]
Partial Scalar^a^	316.91^***^	245	1.29	0.98	0.92	0.98	0.045, [0.029, 0.058]
Means of the latent factors	520.78^***^	249	2.09	0.74	0.58	0.69	0.078, [0.068, 0.087]

*S-B χ^2^, Satorra-Bentler Scaled Statistics; CFI, Comparative Fit Index; NFI, Normed Fit Index; NNFI, Non-Normed Fit Index; RMSEA, Root Mean-Square Error of Approximation; CI, Confidence Interval*.

a *Item intercepts for items 3, 14, and 38 were not constrained*.

* *p < 0.05*,

*** *p < 0.001*.

### Differences between rejected and average students

After confirming the problematic social situations for rejected students, and finding that the model was also explanatory for average students, we determined whether these rejected students' difficulties were greater than those of the control sample made up of students with average sociometric status, and also whether these results were modulated by gender.

Because equality of covariances was not met, Box's *M* = 94.7, *F*_(30, 178301)_ = 3.08, *p* = < 0.001, we used Pillai's trace, as it is the most robust statistic in small samples or when the assumption of covariance homogeneity is violated (Hair et al., 2009; Tabachnick and Fidell, 2013). The multivariate test only revealed differences in the main effects as a function of sociometric status, Pillai's Trace = 0.20, *F*_(4, 331)_ = 20.69, *p* = < 0.001, with a large effect size, $\eta_{p}^{2}$ = 0.20. However, there were no differences as a function of gender, Pillai's Trace = 0.02, *F*_(4, 331)_ = 1.88, *p* = 0.488, $\eta_{p}^{2}$ = 0.02; or in the interaction, Pillai's Trace = 0.01, *F*_(4, 331)_ = 1.13, *p* = 0.260, $\eta_{p}^{2}$ = 0.01.

The inter-subject effects analysis revealed that rejected students' scores were higher than those of average students in all the factors (see Table 8). In the factor Respect for Authority and Rules, the size effect was high. Moderate effects were found in the factors Being Disadvantaged and Prosocial and Empathic Behavior. Finally, the effect size of the factor Response to Own Success was small.

**Table 8:** **Group differences for factor scores between rejected or average sociometric status**.

	**Rejected^a^**		**Average^b^**		***t*_(336)_**	***p***	**Cohen's *d***
	***M***	***SD***	***M***	***SD***			
Being disadvantaged	19.02	5.68	14.73	5.15	7.28	<0.001	0.79
Respect for authority and the rules	8.16	3.27	5.24	2.36	9.38	<0.001	1.02
Response to own success	5.49	2.44	4.46	1.87	4.34	<0.001	0.47
Prosocial and empathic behavior	10.80	4.05	8.38	2.98	7.28	<0.001	0.79

a *n = 169*.

b *n = 169*.

In the case of Prosocial and Empathic Behavior, the Sociometric status × Gender interaction was also significant. In the average group, no differences were found between males (*M* = 8.26, *SD* = 3.05) and females (*M* = 8.61, *SD* = 2.86); *t*_(167)_ = −0.74, *p* = 0.462. However, in the rejected group, males (*M* = 11.26, *SD* = 4.19) had more difficulties than females (*M* = 9.97, *SD* = 3.68); *t*_(167)_ = 2.00, *p* = 0.047, in Prosocial and Empathic Behavior but with a small effect size, *d* = 0.32.

## Discussion

This work aimed to identify the most relevant problematic social situations for rejected students who had just begun elementary school, through the use of the Taxonomy of Problematic Social Situations for Children (TOPS). We found that these situations are related to Being Disadvantaged, Respect for Authority and Rules, Response to their Own Success, and Prosocial and Empathic Behaviors. Rejected children present more difficulties in these situations than their peers—both boys and girls—of average sociometric status. However, within the group of rejected students, boys have more difficulties in Prosocial and Empathic Behavior.

The structural model found for rejected first-grade students presents appropriate fit indices. It is also invariant in the average sample. Our initial hypothesis of a specific structure than that of the original taxonomy was confirmed, like the findings of Matthys et al. (2001) and van der Helm et al. (2013), with whom we coincide in the four-factor structure, but not in some of the typologies and specific behaviors, as we focused on the beginning of compulsory education. Likewise, they consider other situations that, due to the developmental stage, may not be relevant.

The factor that explained the most variance, Being Disadvantaged, corresponds to social situations included in the factors of Peer Group Entry and Response to Provocation from the original taxonomy. In our case, however, in Being Disadvantaged, only the situation in which the student is insulted emerged, but we considered all the intentional and ambiguous provocations, in contrast to Matthys et al. (2001) in Being Disadvantaged or van der Helm et al. (2013) in Disadvantage, where manifest disadvantages were observed, but fewer ambiguous provocations. It must be taken into account that these ages, direct physical aggression is not an isolated behavior although it is highly censored. It may therefore be considered nonproblematic or not exclusive to rejected students, because it is the result of immature behavior, when children are still learning control through self-regulation. This is especially true if the aggression is instrumental. However, this would not occur at later ages where children are expected to have adequate ability to assess the situation and exert the necessary self-control to deal with it in a socially adaptive way. Similarly, problems with provocation, especially ambiguous provocation, are a consequence of rejected students' difficulties to interpret the social signals and characteristics of the context (Dodge et al., 2003; Dirks et al., 2007; Lansford et al., 2010; Asher et al., 2012) and decide whether a provocation is accidental or deliberate.

A second factor we found is the Response to authority and rules, which includes situations relevant to the factor Teacher expectations of the original taxonomy, but only those that specifically involve following rules, both in the presence and absence of the teacher, which coincides with the factor that van der Helm et al. (2013) called Accepting authority. However, unlike the findings of Matthys et al. (2001), being alone on the playground does not discriminate, but being alone in the classroom does. We note that, at these ages, adult supervision and participation in recess is much more intense, and adults tend to be less strict about following rules, as the main goal is for the children to have fun and rest from academic duties. However, in later courses, students are allowed more autonomy to establish and maintain social relations, and they are required to follow the rules, many of which should be internalized. But when they are alone in class, even though this occurs only sporadically and for short periods, it is a prototypical and discriminant situation of rule-following, much clearer than on the playground.

The third factor found, Response to Own Success, coincides with the proposal of Dodge, Response to success. Rejected students have difficulties to identify and regulate emotions, displaying socially maladaptive emotional reactions. No item was considered for the factor of Response to failure. At this age, when competing, many children respond differently from older children. In this sense, other children's success may not necessarily be considered as one's failure, as competitive situations are more diluted than in higher grades, because teachers try to provide all the children with many successful experiences. Other authors grouped these aspects into a single general factor, albeit reduced, related to how children deal with competitive situations, like Coping with Competition (Matthys et al., 2001) or Competition (van der Helm et al., 2013).

The fourth and last factor, Prosocial and Empathic Behavior, slightly corresponds to Social expectations of the original taxonomy. It is similar to Peer expectations (Matthys et al., 2001) and coincides more with that of Accepting/giving help (van der Helm et al., 2013). These results confirm that rejected students have trouble understanding the feelings of others and performing helping behaviors (Bierman, 2004; Bierman et al., 2014), which is very important, as it is one of the characteristics most highly related to social preference (Torrente et al., 2014).

The intensity of rejected students' difficulties in social situations that imply Being Disadvantaged and Respect for Authority and Rules was positively correlated with Antisocial Behavior, especially Hostile, Aggressive, and Disruptive behavior (White and Kistner, 2011). In these social situations, the relation with desirable social competence, such as Self-management/Compliance, is negative. Rose and Asher (2004) noted the importance of paying attention to children's responses to tasks of offering and requesting help.

The lowest, albeit significant, global correlation was between problematic social situations and academic behavior. These results suggest a direct relation between behavioral difficulties and rejection (Bierman, 2004). However, the relation between such difficulties and academic effectiveness is lower at this age, because students are involved in basic learnings, which depend less on effort and dedication than in higher grades.

As mentioned, the structural model obtained in the sample of rejected and average students is invariant except for the means of the latent factors. Consequently, the two groups differ in the intensity of problematic social situations. In fact, all four types of social situations are significantly more problematic for rejected students. Respect for Authority and Rules, followed by Being Disadvantaged—a factor partly made up of items in Response to Provocation—are the most problematic. Like Parker and Asher (1987) and Dodge et al. (1985) agreed that these types of situations were the most difficult for children to master, in particular, for aggressive and rejected children. Next are Prosocial and Empathic Behavior, and lastly, responding to their own success. Rubin and Hubbard (2002) found that rejected children were more likely both to chat and to brag in a game situation.

In terms of gender, within the group of rejected children, rejected boys had more trouble with Prosocial and Empathic Behavior than girls, but not in the other situations. The differences between boys and girls are not caused by the rejection (van Lier et al., 2005) but by manifestation of aggressive and antisocial behavior, more frequent in boys than in girls (Dodge et al., 2003). However, there were no gender differences in the average group.

## Conclusions, limitations and future directions

The study of students' problematic social situations at school opens up many possibilities. Firstly, it can help us to understand the variables involved in social situations at ages that have received little attention with regard to peer rejection. Secondly, because specifically knowing which situations are difficult for rejected students, and differentiating them from those of the average students provides specific intervention guidelines. Hence, interventions would not focus on situations with little discriminant power that could be considered normal for the developmental stage, but on those that differentiate rejected from average students.

In this sense, it is necessary to work on improving students' self-knowledge and self-control, which would give them the skills to follow rules and respect authority. This has a big impact on the formation of their social reputation. We should also enhance emotion identification and regulation to facilitate students' recognizing and emitting the appropriate response to different situations, especially situations involving their own success or intentional and ambiguous provocations. We should not neglect training in empathy, assertiveness, and prosociality, as they are key skills to know how to respond to situations involving disadvantage, how to ask for help but also to offer help adequately, and how to interpret social signals and the characteristics of the situation.

We would have liked to determine whether the model was invariant between boys and girls. However, although the phenomenon of peer rejection has a significant adverse effect on the social development, it must be taken into account that the percentage of identified rejected students per classroom is around 12%. Of them, only 25% are girls. The same thing occurs with the students from other countries or subcultures. This would imply the need to considerably increase the initial sample size and given that, at these ages, data collection is done through individual interviews, it would be very difficult to gather sufficient data.

Finally, it is essential to design and assess intervention proposals to prevent and reduce peer rejection at early ages, contextualized in concrete situations in which rejected students have difficulties. This is important because rejection is not yet chronic at these ages, as the social groups are constantly changing, and the students are learning many of the social skills that can make them resilient to frustration concerning their peers.

## Author contributions

LM: Designed the study, performed the analysis and interpretation of the data, performed the measurement, and draft the manuscript. MM: Contributed to the study design, performed the measurement and participated in drafting the manuscript. FG: Led the study, coordinated data collection, performed the measurement and helped to draft the manuscript. IJ: Contributed to the study design, performed the measurement and helped to draft the manuscript. All authors approved the final manuscript as submitted.

### Conflict of interest statement

The authors declare that the research was conducted in the absence of any commercial or financial relationships that could be construed as a potential conflict of interest.
