# Supplementary material for: Problematic Social Situations for Peer-Rejected Students in the First Year of Elementary School
Source: Front Psychol. 2016 Dec 15;7:1925. doi: 10.3389/fpsyg.2016.01925 (PMC5156692; doi:10.3389/fpsyg.2016.01925)
Supplement: Supplementary file 1 [file DataSheet1.docx]

**Appendix**

Spanish Problematic Social Situations of the Four Factors

| F_1_: Being Disadvantaged [Estar en Desventaja]  6: Cuando los compañeros le insultan  16: Cuando un compañero tiene algo que pertenece a este niño y este niño quiere que se lo devuelva  17: Cuando este niño se da cuenta de que los compañeros le han dejado fuera de un juego, o actividad de compañeros  19: Cuando este niño está jugando con un compañero y el compañero accidentalmente rompe el juguete del niño  24: Cuando este niño es provocado accidentalmente por un compañero (como cuando un compañero tropieza con él estando en la fila)  36: Cuando un compañero expresa su enfado a este niño  F_2_: Respect for Authority and Rules [Respeto a la Autoridad y a las Normas]  27: Cuando el profesor está intentando hablar a toda la clase  28: Cuando este niño está en una fila con sus compañeros y tiene que esperar mucho tiempo  30: Cuando este niño está en clase con sus compañeros y el profesor debe dejar el aula por un corto periodo de tiempo  F_3_: Response to Own Success [Respuesta ante el Propio Éxito]  3: Cuando este niño ha ganado a un compañero en un juego  12: Cuando este niño realiza mejor un juego que un compañero  14: Cuando este niño realiza mejor un trabajo que un compañero  F_4_: Prosocial and Empathic Behavior [Conducta Prosocial y Empática]  33: Cuando este niño está triste y un compañero le pregunta cómo se siente  37: Cuando un compañero ha realizado bastante bien una tarea y se merece y espera un cumplido por parte de este niño  38: Cuando un compañero está preocupado o molesto y espera consuelo por parte de este niño  39: Cuando un compañero ha sido amable y servicial con este niño y éste debería agradecérselo  43: Cuando este niño necesita la ayuda de un compañero y debería pedírsela |
| --- |

*Note*. Item number corresponds with the original TOPS item number (Dodge et al., 1985)
